# Uptake of skilled attendance along the continuum of care in rural Western Kenya: selected analysis from Global Health initiative survey-2012

**DOI:** 10.1186/s12884-018-1803-4

**Published:** 2018-05-16

**Authors:** Winfred Mwangi, Onesmus Gachuno, Meghna Desai, David Obor, Vincent Were, Frank Odhiambo, Amek Nyaguara, Kayla F. Laserson

**Affiliations:** 1 Afya Bora Consortium Fellowship on Global Health Leadership, https://www.afyaboraconsortium.org; 2 Kenya Medical Research Institute/ Centers for Disease Control and Prevention (KEMRI/CDC), Kisumu, Kenya; 3 Moi Teaching and Referral Hospital, Eldoret, Kenya; 4 University of Nairobi, Nairobi, Kenya; 5 Malaria Branch, CDC, Atlanta, Georgia; 6 Centre for Global Health, CDC, Atlanta, Georgia

**Keywords:** Skilled attendance, Maternal and child health, Antenatal care, Newborn care

## Abstract

**Background:**

Examining skilled attendance throughout pregnancy, delivery and immediate postnatal period is proxy indicator on the progress towards reduction of maternal and neonatal mortality in developing countries.

**Methods:**

We conducted a cross-sectional baseline survey of households of mothers with at least 1 child under-5 years in 2012 within the KEMRI/CDC health and demographic surveillance system (HDSS) area in rural western Kenya.

**Results:**

Out of 8260 mother-child pairs, data on antenatal care (ANC) in the most recent pregnancy was obtained for 89% (*n* = 8260); 97% (*n* = 7387) reported attendance. Data on number of ANC visits was available for 89% (*n* = 7140); 52% (*n* = 6335) of mothers reported ≥4 ANC visits. Data on gestation month at first ANC was available for 94% (*n* = 7140) of mothers; 14% (*n* = 6690) reported first visit was in1^st^trimester (0-12 weeks), 73% in 2nd trimester (14-28 weeks) and remaining 13% in third trimester. Forty nine percent (*n* = 8259) of mothers delivered in a Health Facility (HF), 48% at home and 3% en route to HF. Forty percent (*n* = 7140) and 63% (*n* = 4028) of mothers reporting ANC attendance and HF delivery respectively also reported receiving postnatal care (PNC). About 36% (*n* = 8259) of mothers reported newborn assessment (NBA). Sixty eight percent (*n* = 3966) of mothers that delivered at home reported taking newborn for HF check-up, with only 5% (*n* = 2693) doing so within 48 h of delivery. Being ≤34 years (OR 1.8; 95% CI 1.4-2.4) and at least primary education (OR 5.3; 95% CI 1.8-15.3) were significantly associated with ANC attendance. Being ≤34 years (OR 1.7; 95% CI 1.5-2.0), post-secondary vs primary education (OR 10; 95% CI 4.4-23.4), ANC attendance (OR 4.5; 95% CI 3.2-6.1), completing ≥4 ANC visits (OR 2.0; 95% CI 1.8-2.2), were strongly associated with HF delivery. The continuum of care was such that 97% (*n* = 7387) mothers reported ANC attendance, 49% reported both ANC and HF delivery attendance, 34% reported ANC, HF delivery and PNC attendance and only 18% reported ANC, HF delivery, PNC and NBA attendance.

**Conclusion:**

Uptake of services drastically declined from antenatal to postnatal period, along the continuum of care. Age and education were key determinants of uptake.

## Background

The burden of maternal mortality is highest in Sub-Saharan Africa (SSA). Of 287,000 maternal deaths occurring worldwide in 2010, 56% were in SSA [1]. Maternal death rate in Kenya was estimated at 414 (95% CI328-501) in 2003and 488 per 100,000 live births (95% CI333-643) in 2009 [2]. When verbal autopsy methodology is applied the ratio is estimated to be as high as 650 to 669 per 100,000 live births in slums and rural settings [3–5]. Measuring maternal mortality in SSA is difficult and often underestimated. This is due to lack of vital registration systems, wrong attribution of causes of death and because almost 60% of mortality occurs at home [6–8] Skilled birth attendance has been recommended as proxy indicator for maternal deaths [9–12].

The lifetime risk of a Kenyan woman facing maternal death is 1 in 35 yet in 2009 only 44% reported skilled birth attendant [2, 13, 14]. Maternal and newborn deaths can be avoided by ensuring that women receive essential interventions by skilled attendants throughout pregnancy- delivery-postnatal and childhood periods, [15, 16] known as the continuum of care it is designed to ensure continuity of care throughout the woman’s lifecycle. Poor access to effective interventions by needy rural households contributes to preventable deaths [17–20]. Universal access with maternal and newborn key health packages can reduce up to two-thirds of these deaths. Accelerated scale up in SSA is necessary to achieve Millennium Development Goals (MDG) 4 and 5 [18, 20, 21].

Antenatal care (ANC) presents the opportunity for the pregnant woman to receive evidenced-based interventions distributed over 4 individualized visits. The direct contribution of ANC to maternal mortality reduction in developing countries has been questioned due to seemingly successful uptake yet maternal mortality remains high [20–22]. With high prevalence of HIV/AIDS (14%) [2] malaria parasitemia (18%) and anemia in pregnancy (69%) [23] in this area, early ANC allows timely entry into HIV/AIDS and malaria control programs, conditions that substantially compounds maternal mortality and HIV transmission [4, 24].

Skilled birth attendance is the process by which a woman is provided with adequate care during labor, delivery and the early postnatal period. It is the single most effective intervention in reducing maternal and perinatal mortality for two reasons; first, 60% of maternal and 30% of neonatal deaths occur over the short period between labor and the first 24 postnatal hours and second most maternal deaths are due to direct obstetric complications that are unpredictable antenatally but treatable when detected during labor and after birth [7, 25–27]. Thus, location of delivery, who conducts the delivery, and how quickly mothers can be transported to the location where emergency obstetrics care is available are crucial factors to improving the inextricably linked maternal and child survival [28–31].

There are many stakeholders contributing to reproductive health (RH) services in rural western Kenya including the government’s Ministry of Health (MOH), but the usage of RH services that could reduce maternal mortality had not been assessed previously. Due to poor utilization of health facilities, household surveys data provide the next best data estimates that can be used to prioritize resources [6, 12, 32, 33]. Stakeholders under the umbrella of the United States Agency for International Aid (USAID)’s Global Health Initiative (GHI) conducted a population-based survey [34, 35] to assess the uptake of ANC, skilled birth and after delivery services and newborn assessment within 48 h of birth. The objective of this survey was to examine reproductive health service utilization that can be used as indicators to evaluate maternal and child health programs.

## Methods

### Study site and population

The study was conducted from 16th October to 31st December 2012 during the third round of data collection within the KEMRI/CDC’s Health and Demographic Surveillance System (HDSS) area located in Siaya County in rural western Kenya. It has an estimated population of about 220,000 people [36]. Women of reproductive age (15-49 years) make up to 22% of the population and children under-five account for about 16% of the population [36]. The population is predominantly of Luo ethnicity and earns their living through subsistence farming and fishing [37]. HDSS area has high poverty levels and limited healthcare infrastructure. There are 36 health facilities; one district hospital, two privately owned hospitals, 11 health centers and 22 dispensaries [5]. According to the Kenya Essential Package for Health (KEPH) levels of care, Level one consist of the community; Level 2, dispensaries; Level 3, health centers, maternity/nursing homes/ clinics and Level 4, primary hospitals. Level 2 staff include enrolled nurses who can provide antenatal care and treatment for simple medical problems during pregnancy such as anemia, and occasionally conduct normal deliveries [38]. Level 3 is predominantly involved with preventive care, but also various curative services- safe motherhood, child health promotion and integrated management of childhood illnesses (IMCI), Malaria, HIV/AIDS/STI and TB control. Level 4 can handle at least basic emergence obstetric care [34].

The KEMRI/CDC HDSS collects data through house-to-house interviews by trained staff in three rounds every year running from, January to April, May to August, and September to December. Data collected include information on births, deaths, pregnancies, migrations, morbidity, educational and marital status. [37] Malaria transmission is high with seasonal peaks in May-July and October-November. HIV and TB prevalence in HDSS are some of the highest in the country [5].

### Study design

As part of the Global Health Initiative [35] and in collaboration with the Ministry of health (MOH) and other partners in this area in 2011, we conducted a baseline reproductive health house to house survey of mothers with children 5 years and below at the time of interview. All households in the HDSS were targeted. Eligible participants were identified by community interviewers [39] as they visited households in the HDSS as part of the standard HDSS data round. Verbal consent was obtained for these additional service-related questions. As per standard HDSS protocol, individuals were fully aware that participation was voluntary and could opt out at any time during interview. Mothers were asked questions pertaining to the most recent pregnancy; ANC attendance, type of ANC service provider, place of birth. type of birth assistant, after delivery services provided which included PNC, FP and NBA. Data on a Health Facility (HF) check-up for babies born at home was also collected. Postnatal care was defined as service including any assessment given to a mother within 48 h of delivery. The national health policy in Kenya recommends that all women receive postnatal check-up at a HF within 2 days of delivery [2, 13]. PNC can range from simple general exam to and specific exams like blood pressure, breast exam, pain management, mental status exam and Caesarean Section incision site exams. The components and extent of this care is largely dependent on mode of delivery, complications at birth and complaints the mother may present with. Details of the specific service given were not obtained. NBA include early breastfeeding support, umbilical cord hygiene, keeping baby warm and immunization and identifying danger signs for severe illness like breathing difficulties, lethargy and inability to feed [20].

The child’s mother was interviewed; where the mother was not present at the time of interview, a proxy (the head of household or household administrator) was interviewed. Household administrator is the person who runs the household day to day affairs on behalf of a non-resident head of household [39]. When the mother or proxy was unavailable on a second visit, the household was excluded from the survey. Socio-demographic data collected a year prior was obtained from the HDSS database using the mothers’ Permanent Identification number issued by HDSS and useful in linking records of individuals across files [39].

### Data analysis

This study employed quantitative analysis. Responses reported as “Don’t Know” or “NA” were excluded from analysis. Both descriptive and inferential analysis was done using Epi info. Continuous variables were described by medians, interquartile ranges, and standard deviations. Categorical variables were described as proportions. Associations for categorical data were determined using Pearson’s chi-square test or Fisher’s exact test. We evaluated factors associated with ANC, skilled birth attendance, PNC and NBA through bivariate analyses, using logistic regression. All tests were interpreted against a 5% significance level.

## Results

### Socio-demographic characteristics

We enrolled 8377 mothers with at least one child who was under five at the time of interview. Using the HDSS permanent ID we linked mothers’ data with the youngest child and obtained 8260 mother-child pairs. Because of missing data the denominator for each variable was different. The median age of mothers was 27 years (range: 13-54 years) (Table 1).

**Table 1:** Socio-demographic and clinical Characteristics

	Number	Percent	95% CI	
Age Category *n* = 8096				
≤ 20	1063	13.1%	12.4	13.9
21-34	5571	68.8%	67.8	69.8
≥ 35	1462	18.1%	17.2	18.9
Marital Status *n* = 7788				
Married	3994	51.3%	50.2	52.4
Married/Cohabiting	2176	27.9%	27	29
Divorced/Separated	128	1.6%	1.4	2
Widowed	280	3.6%	3.2	4
Single	1210	15.5%	14.7	16.4
Level of Education *n* = 3887				
Primary	2966	76.3%	74.9	77.6
Secondary	812	20.9%	19.6	22.2
Post-secondary	73	1.9%	1.5	2.4
None	36	0.9%	0.7	1.3
ANC attendance (at least one visit) *n* = 7387				
Yes	7140	96.7%	96.2	97.1
No	247	3.3%	3	3.8
Gestation month at first ANC visit *n* = 6696				
1-3	945	14.1%	13.3	15
4-6	4882	72.9%	71.8	74
7-9	869	13.0%	12.3	13.8
Number of ANC visits made *n* = 6332				
1	231	3.7%	3.2	4.2
2	866	13.7%	12.8	14.6
3	1957	30.9%	29.8	32.1
4	1905	30.1%	29	31.2
5	868	13.7%	12.9	14.6
≥ 6	505	8.0%	6.9	9.3
Source of ANC *n* = 7119				
Government Dispensary	2014	28.3%	27.3	29.4
Government Health Centre	2583	36.4%	35.2	37.4
Government Hospital	1703	23.9%	22.9	24.9
Mission Hospital	534	7.5%	6.9	8.1
Private Clinic/Hospital	236	3.3%	2.9	3.8
Nursing/Maternity Home	7	0.1%	0	0.2
Home	36	0.5%	0.4	0.7
Others	6	0.1%	0	0.2
ANC service provider *n* = 7140				
Doctor	651	9.1%	8.5	9.8
Nurse/Midwife	6351	89.0%	88.2	90
Community health workers	41	0.6%	0.4	0.8
Traditional birth attendant	52	0.7%	0.6	1
Others	45	0.6%	0.5	0.9
Birthplace *n* = 8259				
Government Dispensary	529	6.4%	5.9	7
Government Health Centre	1038	12.6%	11.9	13.3
Government Hospital	1807	21.9%	21	22.8
Mission Hospital	405	4.9%	4.4	5.4
Private Clinic/Hospital	211	2.6%	2.2	2.9
Nursing/Maternity Home	38	0.5%	0.3	0.6
Home Delivery	3970	48.1%	47	49.1
En route to Health facility	261	3.2%	2.8	3.6
Birth Assistants *n* = 8257				
Doctor	574	7.0%	6.4	7.5
First Nurse then doctor	547	6.6%	6.1	7.2
Nurse/midwife	3202	38.8%	37.7	39.8
Community Health Worker (CHW)	1008	12.2%	11.5	12.9
Traditional Birth Attendant (TBA)	1610	19.5%	18.7	20.4
Others(relatives, friends, neighbours, self)	1316	15.9%	15.2	16.8
After delivery service (Following HF delivery) *n* = 4028				
Postnatal care	2554	63.4%	61.9	64.9
FP counselling	1349	33.5%	32	35
Assessment of newborn	2373	58.9%	57.4	60.4
Newborn HF check-up following home delivery *n* = 2693				
Same day	8	0.3%		
day one	43	1.6%		
day two	62	2.3%		
day 3	75	2.8%		
day 4	61	2.3%		
day 5	39	1.5%		
day 6	43	1.6%		
day 7	423	15.7%		
≥ 8	1939	72.0%		

Denominators vary for each variable depending on missing data

### Antenatal care

Complete data on ANC attendance was available for 89% (*n* = 8260) of mothers, 97% (*n* = 7387) of whom reported attending ANC. Data on number of ANC visits made was available for 89% (*n* = 7140); 4% (*n* = 6335) of mothers reported making only one ANC visit, 14% 2 visits, 31% 3 visits, 30% 4 visits, 14% 5 visits and the remaining 8% reported ≥6 ANC visits. Only 52% (*n* = 6335) of mothers completed the WHO recommended 4 ANC visits. Data on gestation month at first ANC visit was available for 94% (7140) of mothers; 14% (*n* = 6690) reported first ANC visit was in1sttrimester (0-12 weeks), 73% in 2ndtrimester (14-28 weeks) and 13% in 3rdtrimester (29-40 weeks) (Table 1). The median number of gestation months at first visit was 5. Skilled ANC service provider was reported by 98% (*n* = 7140) of mothers: 91% (*n* = 7002) a nurse/midwife and 9% a doctor. Non-skilled ANC care providers were reported as TBAs by 38% (*n* = 138), CHWs by 30% and “others” specified as co-wives, neighbors or relatives were reported by 33% of mothers.

**Fig. 1:**
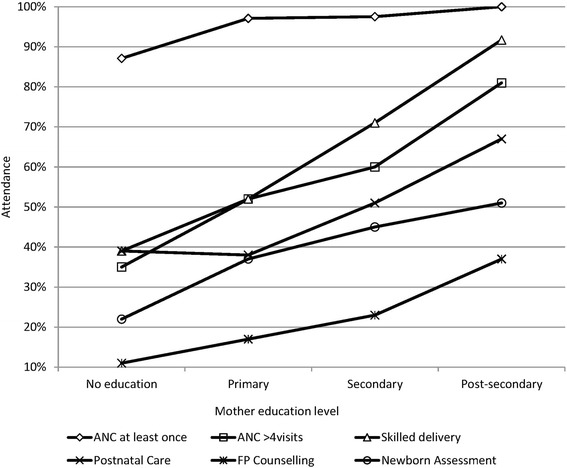
Uptake of services according to education level

The proportion of mothers attending ANC at least once and those making ≥4 ANC visits increased with level of education: 100% (*n* = 67) and 83% (*n* = 48) of mothers with post-secondary education and 87% (*n* = 31) and 44% (*n* = 9) with no education reported ANC attendance and completed ≥4 ANC visits respectively (Fig. 1).

**Fig. 2:**
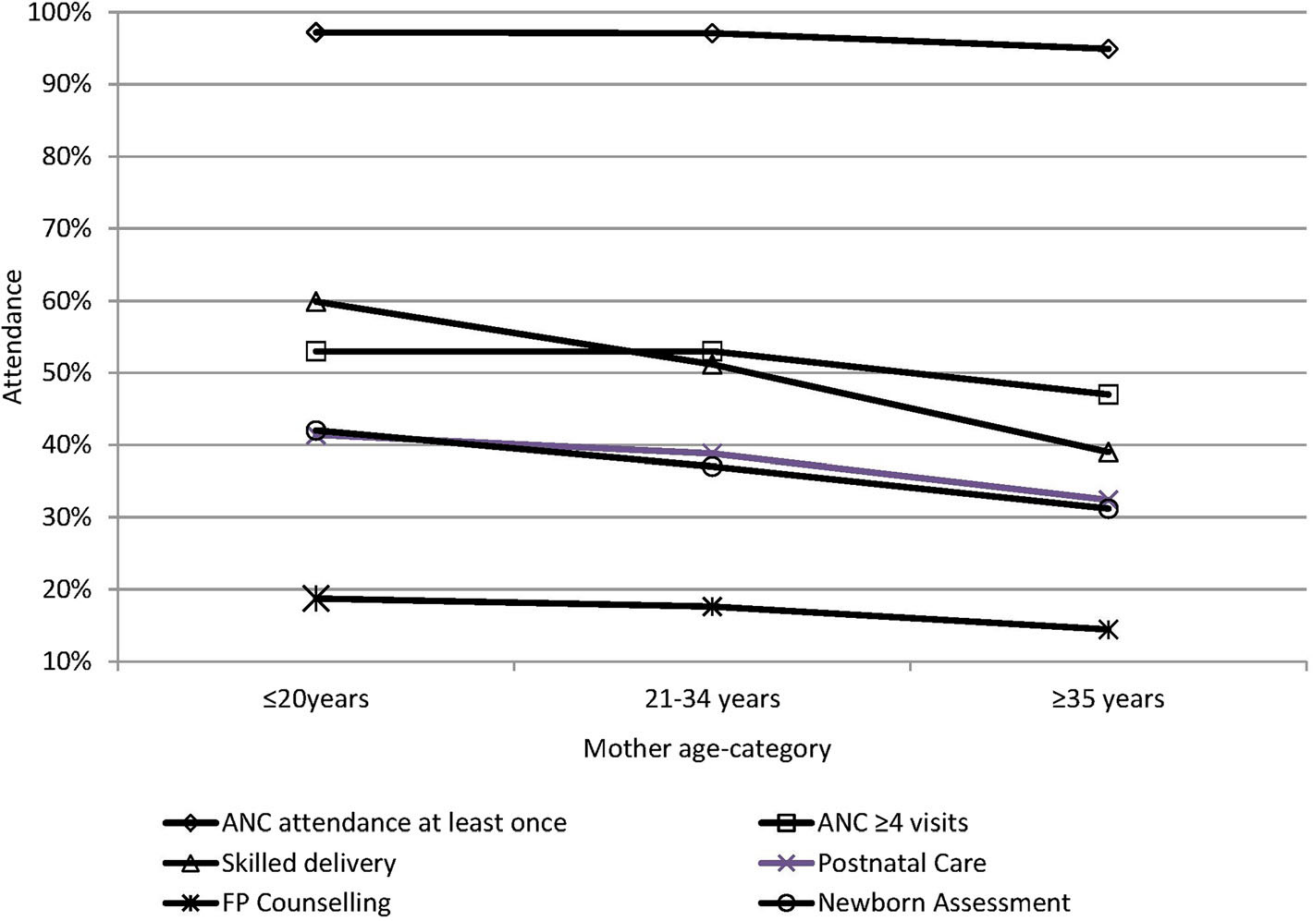
Uptake of services according to age-category

On the other hand, the percentage of mothers attending ANC at least once and those making ≥4 ANC visits decreased with age: 97% (*n* = 951) and 53% (*n* = 835) of mothers aged ≤20 years reported ANC attendance and completed ≥4 ANC visits respectively and 95% (*n* = 1337) and 47% (*n* = 1091) respectively of mothers aged ≥35 years (Fig. 2).

**Fig. 3:**
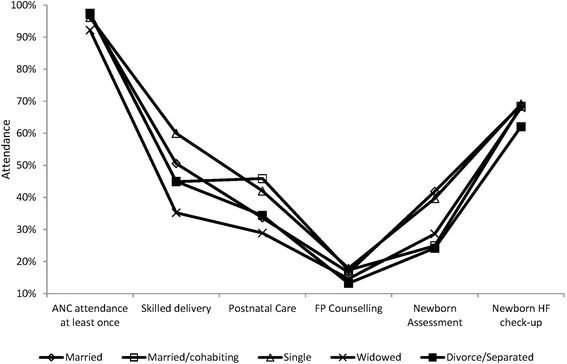
Uptake of services according to marital status

ANC attendance and completion of ≥4 ANC visits by marital status is shown in Fig. 3.

Being ≤34 years (OR 1.8; 95% CI1.4-2.4), any level vs no education (OR 5.3; 95% CI1.8-15.3), primary vs no education (OR 5.0; 95% CI 1.7-14.6), secondary vs no education (OR 5.8; 95% CI 1.8-18.4), married vs widowed (OR2.4; 95% CI1.5-3.9), married vs divorced/separated (OR 1.7; 95% CI 0.9-3.6) were significantly associated with increased ANC attendance (Table 2). Young age ≤ 20 vs ≥35 years (OR 1.7; 95% CI 1.2-2.4) and secondary vs primary education (OR 1.5; 95% CI 1.1-2.0) were significantly associated with early ANC- in first or second trimester.

**Table 2:** Relationship between socio-demographic characteristics and antenatal care attendance

Attended ANC		Yes *(n)*		No *(n)*		Odds Ratio (95% CI)	*P*-value
Marital status	[Married/Married & cohabiting] / [Single/Divorced/Separated/widowed]	5452	96.9%	173	3.1%	1.5(1.1-2.0)	0.02^*^
		1337	95.6%	62	4.4%		
	Married / Widowed	3475	96.6%	123	3.4%	2.4(1.5-3.9*)*	0.001^*^
		235	92.2%	20	7.8%		
Level of Education	At least primary level / None at all	3334	97.2%	94	2.8%	5.3(1.8- 15.3)	0.01^*^
		27	87.1%	4	12.9%		
		707	97.5%	18	2.5%	5.8 (1.8-18.4)	0.01^*^
	Secondary/ no education	27	87.1%	4	12.9%		
	Primary/ No education	2560	97.1%	76	2.9%	5.0 (1.7-14.6)	0.01^*^
		27	87.1%	4	12.9%		
Mother’s age-category	≤20 years / ≥21 years	901	97.1%	27	2.9%	1.1(0.8-1.2)	0.8^*^
		6026	96.5%	216	3.5%		
	21-34 / ≥35 years	4865	97.1%	147	2.9%	1.8 (1.3-2.4)	0.001^*^
		1269	94.9%	68	5.1%		
	≤20 / ≥35 years	924	97.2%	27	2.8%	1.8 (1.2-2.9)	0.01^*^
		1269	94.9%	68	5.1%		
	≤34 / ≥35 years	5789	97.1%	174	2.9%	1.8 (1.4-2.4)	0.001^*^
		1270	94.9%	69	5.2%		

Reference: ^*^Fischer’s Exact

Denominators vary for each variable depending on missing data

### Delivery care

Only 49% (*n* = 8259) of mothers delivered in a HF, 48% at home, and the remaining 3% en route to a HF (Table 1). Of all mothers who reported a HF delivery, 84% (*n* = 4028) utilized a government-owned HF while the remaining delivered in a private HF. Types of government-owned facilities utilized for delivery included; Hospitals by 54% (1807), health centers by 31% (1038) and dispensaries by 16% (529). Types of private HFs utilized for delivery included; Mission Hospital by 62% (405), Private Hospitals/Clinics by 32% (211) and Nursing & Maternity Home by 6% (38) of mothers. Government hospital was the most utilized facility for delivery. Skilled birth attendants at delivery was reported by 52% (*n* = 8257) of mothers while the remaining 48% reported a non-skilled birth attendant. Skilled birth attendants were reported as nurse/midwife in 87% (*n* = 4323) and doctor in 13%. Non-skilled attendants were reported as TBAs by 41% (*n* = 3934), CHWs by 26% and other types of birth assistants by 33%. The “other types” were specified as; friends or neighbors 15% (*n* = 1316), relatives 12%, mother delivered on her own in 53% and in 20% data was missing (Table 1). The relatives were reported as sisters, co-wives or mother-in-law. Almost all the births assisted by CHWs and TBAs happened at home or en route to a HF, 94% (*n* = 1008) and 99% (*n* = 1610) respectively. However, 59 mothers assisted by CHW and 51 by TBA reported HF delivery. We did not evaluate whether these were mothers who started off with non-skilled birth attendant and were later referred to a HF or not. Likewise 3% (*n* = 4028) of mothers reporting HF delivery, reported a non-skilled birth assistant which may indicate mothers’ inability to identify the cadre of health service provider. Similarly, 11% (*n* = 3968) of mothers reporting home delivery, reported a skilled birth assistant.

The proportion of mothers reporting a HF delivery increased with the level of education and decreased with the age (Figs. 1 and 2). HF delivery according to marital status is shown in Fig. 3. Young age ≤ 34 years (OR 1.7; 95% CI1.5-2.0), Secondary vs primary education (OR 2.2; 95% CI1.8-2.6), post-secondary vs secondary education (OR 4.6; (95% CI2.0-10.8), post-secondary vs primary education (OR10.1; 95% CI4.4-23), single vs married, (OR 1.5; 95% CI1.3-1.7), single vs widowed, (OR 2.8; 95% CI2.1-3.6) were significantly associated with a HF delivery (Table 3).

**Table 3:** Relationship between socio-demographic characteristics, antenatal care attendance and the place of delivery

Type of delivery		HF Delivery *(n)*		Home delivery *n)*		Odd Ratio (95% CI)	*P*-value
ANC attendance	Yes / No	3623	51.4%	3427	48.6%	4.5 (3.2-6.1)	0.001^*^
		47	19.1%	198	5.5%		
ANC place	HF vs Home	3598	51.5%	3390	49.0%	2.9 (1.4-5.8)	0.002^*^
		11	26.8%	30	73.2%		
ANC provider	Skilled/non-skilled	3568	51.5%	3349	48.4%	1.5 (1.1-2.1)	0.02^*^
		55	41.2%	78	58.7%		
Gestation at first visit	1-3 months / 7-9 months	597	63.7%	340	36.3%	3.0 (2.5-3.7)	0.001^#^
		311	36.8%	535	63.2%		
	1-3 months / 4-6 months	597	63.7%	340	36.3%	1.6 (1.4-1.9)	0.001^#^
		2463	51.3%	2335	48.7%		
	4-6 months / 7-9 months	2463	51.3%	2335	48.7%	1.8 (1.6-2.2)	0.001^*^
		311	36.8%	535	63.2%		
	1-6 months /7-9 months	3083	53.5%	2675	46.5%	1.9 (1.7-2.2)	0.001^*^
		318	37.3%	535	62.7%		
Number of ANC visits	≥4 / ≤3 visits	1899	55.6%	1341	41.4%	1.8 (1.7-2.1)	0.001^#^
		1298	43.1%	1714	56.9%		
Education	At least Primary level / None	2122	57.0%	1602	43.0%	2.1 (1.1-4.1)	0.04^*^
		14	38.9%	22	61.1%		
	Post-secondary / Primary	66	91.7%	6	8.3%	10.1 (4.4-23.4)	0.001^*^
		1485	52.1%	1363	47.9%		
	Post-secondary / Secondary	66	91.7%	6	8.3%	4.6 (2.0-10.8)	0.001^*^
		553	70.5%	232	29.65		
	Secondary / Primary	556	70.6%	232	29.4%	2.2 (1.8-2.6)	0.001^#^
		1500	52.4%	1363	47.6%		
Marital status	Married / Widowed	1955	50.6%	1906	49.4%	1.9 (1.5-2.4)	0.0001^*^
		95	35.2%	175	64.8%		
	Single / Widowed	695	59.7%	469	40.3%	2.8 (2.1-3.6)	0.001^*^
		94	34.9%	175	65.1%		
	Married / [Married/Cohabiting]	1955	50.6%	1906	49.4%	1.3 (1.1-1.4)	0.001^#^
		958	44.9%	1174	55.1%		
	Single / Married	695	59.7%	469	40.3%	1.5 (1.3-1.7)	0.001^#^
		1929	50.3%	1906	49.7%		
Mother’s Age	< 20 years / ≥20 years	418	60.1%	277	39.9%	1.6 (1.3-1.8)	0.001^*^
		3487	48.9%	3641	51.1%		
	≤34 / ≥35	3386	52.6%	3047	47.4%	1.7 (1.5-2.0)	0.001^#^
		556	39.0%	871	61.0%		
	≤ 20 / ≥ 35 years	612	59.7%	413	40.3%	2.3 (2.0-2.8)	0.001^#^
		551	38.8%	871	61.3%		
Type of birth assistant		Skilled		Non-skilled			
ANC place	HF /Home	3864	54.6%	3211	45.4%	2.2 (1.2-4.1)	0.02^*^
		15	35.7%	182	64.3%		
Number of ANC visits	≥4 / ≤3 visits	2063	62.9%	1218	37.1%	2.0 (1.8-2.2)	0.001^#^
		1395	45.7%	1657	54.3%		
Gestation at 1st visit	4-6 months / 7-9 months	2691	55.1%	2190	44.9%	1.8 (1.6-2.2)	0.001^*^
		344	39.6%	524	60.4%		
ANC provider	Skilled / non-skilled	3850	55.0%	3150	45.0%	2.8 (1.9-4.0)	0.001^*^
		42	30.4%	96	69.6%		
Education	Post-secondary/ primary education	64	87.7%	9	12.3%	5.9 (2.9-12.0)	0.001^*^
		1615	54.5%	1350	45.5%		

Reference: ^*^Fischer’s Exact, ^#^Chi Square

Denominators vary for each variable depending on missing data

About 51% (*n* = 7140) of mothers who reported ANC attendance also reported a HF delivery. Nineteen percent (*n* = 245) of women reporting no ANC reported a HF delivery. ANC variables strongly associated with a HF delivery included; ANC attendance (OR 4.5; 95% CI 3.2-6.1), HF as source of ANC (OR 2.9; 95% CI 1.4-5.8), first visit in 1st or 2nd vs 3rd trimester (OR 1.9; 95% CI1.7-2.2), first visit in 1st vs 3rd trimester (OR 3.0; 95% CI 2.5-3.7) and having completed ≥4 ANC visits (OR1.8; 95% CI1.7-2.1). There was a strong relationship between reporting skilled ANC provider (OR 2.8; 95% CI 1.9-4.0), first visit during 2nd vs 3rd trimester (OR1.8; 95% CI1.6-2.2), completing ≥4visits (OR 2.0; 95% CI 1.8-2.2), post-secondary vs primary education (OR 5.9; 95% CI2.9-12.0) and reporting a skilled birth assistant (Table 3).

### After-delivery services

We asked mothers whether they received any after delivery service namely, PNC and FP within 48 h of delivery. About 38% (*n* = 8260) and 17% of mothers reported to have received PNC and FP respectively. Only 40% (*n* = 7140) and 63% (*n* = 4028) of mothers who reported ANC and HF delivery respectively, also reported receiving PNC (Table 1). Of mothers who delivered away from HF, only 14% (*n* = 4232) reported receiving PNC. Thirty four percent (*n* = 4028) of mothers who reported a HF delivery and a mere 1% (*n* = 4232) of those who delivered away from HF also reported receiving FP. The proportion of mothers reporting PNC or FP increased with level of education and decreased with age. According to level of education: 67 and 37% (*n* = 73) of mothers with post-secondary education reported PNC and FP attendance respectively, 51 and 23% (*n* = 812) with secondary education, 38 and 17% (*n* = 2966) with primary education and 39 and 11% (*n* = 36) with no education. According to age: 41 and 19% (*n* = 1063) of mothers aged ≤20 years reported PNC and FP respectively; 39 and 18% (*n* = 5571) aged 21-34 years; 32 and 14% (*n* = 1462) aged ≥35 years. Despite coming into contact with health care worker 37% (*n* = 4028) and 67% (*n* = 4028) of mothers reported not receiving any PNC or FP respectively.

Young age ≤ 20 years (OR 1.5; 95% CI 1.2-1.7), secondary vs primary education (OR 1.7; 95% CI1.4-2.0), ANC attendance (OR 3.4; 95% CI2.4-4.8) and HF delivery (OR11; 95% CI 10-12) were significantly associated with increased uptake of PNC (Table 4). Mothers who delivered in a HF were more likely to receive FP compared with those who delivered away from HF, (OR34; 95% CI26-44) *p-value = 0.001*.

**Table 4:** Relationship between socio-demographic characteristics and postnatal and the newborn care

		Yes (n*)*		No *(n)*		Odd Ratio (95% CI)	*P*-value
Received postnatal care							
ANC attendance	Yes /No	2882	40.4%	4258	59.6%	3.4 (2.4-4.8)	*0.001* ^*^
		41	16.6%	206	83.4%		
Birth Where	HF / Home	2554	63.4%	1474	36.6%	10.6 (9.5-11.9)	*0.001* ^*#*^
		555	14.0%	3415	86.0%		
Level of education	Secondary /Primary	411	50.6%	401	49.4%	1.7 (1.4-2.0)	*0.001* ^*^
		1126	38.0%	1840	62.0%		
Mothers age	< 20 years / > 35 years	440	41.4%	623	58.6%	1.5 (1.2-1.7)	*0.001* ^*^
		474	32.4%	988	67.6%		
Received newborn assessment							
ANC attendance	Yes/ No	2676	37.5%	4464	63.0%	2.4 (1.7-3.2)	*0.001* ^*^
		50	20.0%	197	80.0		
Birth place	HF / Home	2373	58.9%	1655	41.1%	7.8 (7.0-8.7)	*0.001* ^*#*^
		616	15.5%	3354	84.5%		
Level of education	At least primary level/ none	1492	40.1%	2231	59.9%	2.3(1.1-5.1)	*0.04* ^*^
		8	22.2%	28	77.8%		
Received newborn Health Facility check-up							
ANC attendance	Yes / No	2396	70.0%	1029	30.0%	1.6(1.2-2.1)	*0.003* ^*^
		118	59.6%	80	40.4%		
Birth place	HF/Home	2112	61.3%	1331	38.7%	1.4 (1.3-1.6)	*0.001* ^*#*^
		1815	52.3%	1653	47.7%		
Level of education	At least primary level /none	1094	68.5%	504	31.5%	1.5(0.6-3.5)	*0.36* ^*^
		13	59.1%	9	40.9%		

Reference: ^*^Fischer’s Exact, ^#^Chi Square

Denominators vary for each variable depending on missing data

### Newborn care

We enquired from mothers if their newborns were assessed within 48 h after delivery. About 36% (*n* = 8259) of mothers reported NBA, 79% (*n* = 3007) of whom reported a HF delivery, 20% home delivery and 1% en route to HF. Only 37% (*n* = 7140) of the mothers who reported ANC attendance and 59% (*n* = 4028) who reported a HF delivery also reported NBA. Just like with PNC, the proportion of mothers reporting NBA increased with level of education and decreased with age. ANC attendance (OR 2.4; 95% CI 1.7-3.2), HF delivery (OR 7.8; 95% CI7.0-8.7) and having education vs none (OR 2.3; 95% CI 1.1-5.1) were significantly associated with reporting NBA (Table 4). Despite reporting a HF delivery, 41% (*n* = 4028) of mothers reported no NBA.

Sixty eight percent (*n* = 3966) of mothers who delivered at home, reported taking the newborn for a HF check-up. Less than 1 %, 0.3% (*n* = 2697) took the newborns for HF check-up the same day they were born, 1.6% were taken the following day, 28% within the first week, 77% by 2 weeks. Mean duration within which newborns were taken to a HF for check-up was 44 days. ANC attendance (OR 1.6; 95% CI 1.2-2.1) and HF delivery (OR 1.4; 95% CI 1.3-1.6) were significantly associated with increased likelihood of reporting a HF check-up (Table 4).

### Continuum of care

**Fig. 4:**
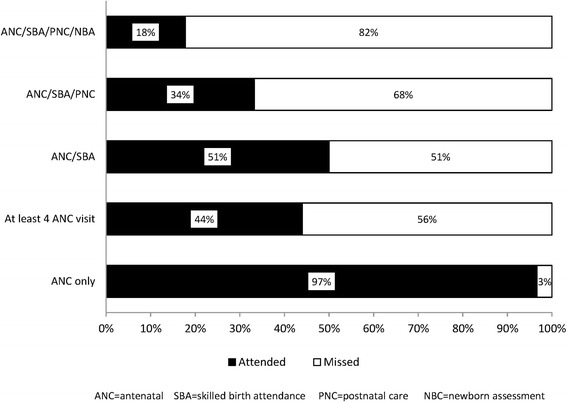
Percent coverage of essential interventions along the continuum of care

About 97% (*n* = 7387) of mothers reported having attended ANC during their most recent pregnancy, 51% reported both ANC attendance and HF delivery, 34% reported ANC, HF delivery and PNC attendance. Mothers reporting all services along the continuum of care; ANC attendance, HF delivery, PNC and NBA assessment were only 18% (Fig. 4).

## Discussion

This study reports the uptake of maternal and newborn health services. These can serve as health services utilization indicators for maternal and newborn health in western Kenya. This has a two-fold benefit. First, we provide baseline indicators especially useful for monitoring and evaluation of programs. Secondly, it addresses the paucity of newborn care indicators: the Kenya essential care package has only two indicators on newborn care –Bacille Calmette Guerin (BCG) vaccination and newborn resuscitation [34]. The Kenya devolved government from national to county level and these indicators estimates presents an opportunity for rural counties to design programs that are more responsive to local needs in order to reduce disparities in Health care. We found high ANC coverage of 97%, above the national figure of 94% reported in 2009 [2]. The pattern of ANC attendance according to gestation month correlates with results from other studies done in this area in 2002 [40] and in most of SSA [30, 37, 41, 42], where majority initiated ANC in second trimester and only about half completed 4 ANC visits. Therefore, despite high ANC coverage in Africa, this does not always translate to quality care, a pattern observed in previous studies [2, 41, 43]. Early attendance and completion of ≥4 ANC was associated with HF delivery. The effectiveness of interventions such, Intermittent Preventive Therapy (IPT) for malaria and prevention of mother-to-child transmission (PMTCT) of HIV depend on early and repeated ANC visits [44]. To achieve the full benefits of ANC, at least four visits of focused antenatal care distributed over all the trimesters are necessary and early attendance need to be encouraged [30, 45].

The findings in this survey confirm the trends observed in other studies- low uptake of skilled birth services [45–47] skilled birth attendance in terms of HF delivery and skilled birth assistant was 49 and 52% respectively, far below the 90% target by 2015 [2, 13, 47]. Low-income countries that have reported major reductions in maternal and neonatal mortality have also reported high skilled birth attendance [48]. A third of women delivered under the care of CHW and TBAs almost 10 years after shift in government policy to use of skilled birth attendants instead of trained TBA [49]. Since 2003, government policy was that all deliveries in Kenya should be conducted by skilled birth attendants even at the community level. However studies done in Kenya, on factors influencing uptake of skilled birth recommend change of policy [46, 50]. Policy documents indicate that the cadres of health workers to offer skilled birth at the community level under the Community Health Strategy is under consideration [13, 34]. Of concern also is the finding that about 8% (617) of mothers reported giving birth all alone, in the event of the ever so common postnatal hemorrhage they are at an especially high risk of death [4]. About 11% (428) reported a skilled birth assistant. Home-based skilled birth has been observed elsewhere in Kenya [51]. This strategy can increase coverage of skilled birth attendance in rural areas and has worked well countries in like the Netherlands [24, 25]. However the lack of equipment and supplies and the short duration within which emergencies must be tackled, can render even the most skilled attendant unable to cope.

Maternal age was a determinant of use of both ANC and HF delivery as reported in other studies [47]. Mothers ≥35 years were more likely not to attend ANC, to attend late and to deliver at home and therefore at highest risk of maternal mortality [52, 53]. Younger mothers fear of complications at childbirth and therefore opt for HF delivery. Older mothers often have had a previous normal delivery and are unwary of emergencies that could occur at birth [46, 47, 54]. Uptake of skilled birth by younger mothers just like with ANC was higher compared to mothers aged ≥35 years. One study in 1999 found teenagers to be more likely to deliver at home but our data as well as that of more recent studies indicate the contrary [47, 55].

Education was not only a strong predictor of uptake of skilled birth services as shown in other studies [40, 52], but we found it was associated with increased uptake of all the services we examined in this study. Girl education even when it is just basic education has a positive impact on uptake of maternal services. Mothers in a marital relationship were more likely to attend ANC compared to those who were not as reported in one study [47]. When it came to delivery single women were most likely to reported HF delivery. Compared to married women single mothers have greater autonomy as they may not have to depend on decisions of husbands and/or mothers-in-laws [45, 47]. The proportion of widowed mothers attending ANC and skilled birth was the lowest. Our findings agree with similar data in SSA where, age, marital status and education were associated with increased ANC attendance [45, 56].

Data from the 90s showed postnatal care (PNC) in developing countries is almost non-existent [57]. Our data showed that six in ten mothers did not receive PNC. Nevertheless, mothers who delivered in HF were 11 times more likely to receive PNC compared with those who delivered away from HF. Lack of a defined routine postnatal care package has been reported as one hindrances to services delivery at HF level [29, 57, 58]. Every mother who delivers at HF and is discharged without PNC and/or FP counseling represents an unfortunate missed opportunity for service [30].

Mothers who delivered at home in the first instance were least likely to report uptake of after-delivery services. In rural settings where many mothers deliver at home, instead of assuming that the mother or baby will be brought to HF, integrated postnatal home-visit packages for mother and newborn by appropriate health workers supported with linkage to referral care are a viable alternative [24, 29].

The community health strategy (CHS) is one innovative way adopted in Kenya to improve maternal and newborn services delivery [59]. CHS prioritization shall be based on maternal, newborn and child health indicators and hard-to–reach communities. Our data identifies HDSS as an underserved population and would benefit from such a strategy [14].

We would like to highlight our assumptions in this study that mothers could correctly distinguish between the different cadres of health service providers and that the HFs were an enabling environment in terms of adequacy of trained personnel, supplies and equipment and that there was a functioning referral system. The limitations in this study are inherent to population-based studies [60]. We relied on self-reported data based on the interviewee’s responses which were not observed. Questions were about events that could have taken place 2 weeks to 5 years ago and this may have introduced recall bias. About 99% of mothers interviewed reported pregnancies that occurred in the 3 years preceding the survey which may have minimized bias.

## Conclusion

We have identified key maternal and newborn health indicators that address the paucity, particularly of PNC and newborn care indicators and provided baseline indicators that can be used for developing new and evaluation of existing maternal and child health programs. The findings in this survey confirm the trends observed in other studies in Africa that there is high uptake of antenatal care but low uptake of subsequent skilled services along the continuum of care. There were missed opportunities to offer PNC, FP and newborn care to mothers who managed to reach HF. Young age and having education were associated with increased uptake of all services while being widowed was associated with poor uptake of services. Over half of mothers delivered away from HF and were less likely to receive after delivery services innovative approaches targeting this group are needed.

## Abbreviations

AIDS: Acquired immune deficiency syndrome
ANC: Antenatal care
BCG: Bacillus calmette–guérin
CDC: Centre for disease control
CHS: Community Health Strategy
CHW: Community Health Worker
CI: Confidence interval
FP: Family planning
GHI: Global Health Initiative
HDSS: Health Demographic Surveillance System
HF: Health facility
HIV: Human Immunodeficiency Syndrome
IMCI: Integrated Management of Childhood Illnesses
IPT: Intermittent Preventive Treatment
KEMRI: Kenya Medical research Institute
KEPH: Kenya Essential Package for Health
MDG: Millennium Development Goals
MOH: Ministry of health
NBA: Newborn assessment
OR: Odds ratio
PMTCT: Prevention of Mother to Child Transmission
PNC: Postnatal care
RH: Reproductive health
SERU: Scientific and Ethics Review Unit
SSA: Sub-Saharan Africa
STI: Sexually Transmitted Infections
TB: Tuberculosis
TBA: Traditional Birth Attendant
USAID: United States Agency for International Aid
